# Traumatic Brain Injury: Patient Characteristics, Hospital Costs and Trends Over Time

**DOI:** 10.36469/9893

**Published:** 2014-10-01

**Authors:** Pankaj A. Patel, Peter J. Mallow, Mary Vassar, John A. Rizzo, Bhavik J. Pandya, Denise T. Kruzikas

**Affiliations:** 1 GE Healthcare, Barrington, IL, USA; 2 CTI Clinical Trial and Consulting Services, Cincinnati, OH, USA; 3 University of California Brain and Spinal Injury Center, San Francisco, CA, USA; 4 Stony Brook University, Stony Brook, NY, USA

**Keywords:** inpatient admission, patient outcomes, rates, hospital costs, traumatic brain injury

## Abstract

**Background:** Traumatic brain injury (TBI) is an increasingly diagnosed condition, but the trends in TBI visits and the cost of which have not been quantified from the hospital perspective.

**Objectives:** To quantify the costs of TBI stratified by inpatient and outpatient visits and to examine trends in TBI incidence over time.

**Methods:** This descriptive study utilized data for 2007-2012 from the Premier hospital database, which includes clinical and utilization information from hospitals across the United States. TBI was identified through International Classification of Diseases, 9th Revision, Clinical Modification (ICD-9-CM) codes. Descriptive data were obtained to identify the TBI costs, visit costs, patient characteristics, and intertemporal trends in TBI rates.

**Results:** TBI patients were treated on an outpatient basis 88% of the time. Nearly 45% (44.3%) of TBI patients requiring inpatient admissions were age 65 or over, and 20% of TBI patients treated as an outpatient were age 75 or over. Children aged 4 or younger accounted for nearly 14% of TBI cases treated on an outpatient basis. TBI patients treated in the inpatient setting incurred fairly long hospital visits (mean 4.8 days; median 3.0 days) and substantial hospital costs (mean $12,717; median $8,176). The rate of TBI visits have risen substantially over time, especially among children under age 18 years and patients in the Northeast US Census Region.

**Conclusion:** TBI is a serious medical condition that appears to be on the rise. Large differences exist between the hospital costs associated with TBIs treated in the inpatient and outpatient settings. Further research to understand factors affecting the costs and clinical outcomes of TBI can help refine treatment strategies to enhance patient outcomes while providing cost effective care.

### INTRODUCTION

Traumatic brain injury (TBI) is common in the United States, with an estimated 1.7 million occurrences each year.[1] According to a 2010 Centers Disease Control and Prevention (CDC) report, TBI results in 275,000 hospitalizations and 52,000 deaths annually.[1] Annual costs attributed to TBI are substantial in the United States, with an estimated $76.5 billion in direct medical costs and indirect costs, such as lost productivity and wages.[1,2]

Given the impact of TBI, more research has focused on this topic. A recent survey of US Levels 1 - 3 trauma centers indicated dramatic improvement in adherence to Brain Trauma Foundation guidelines for management of patients with severe TBI in contrast to earlier survey evidence.[3] Hesdorffer et al.[3] commented on the importance of adhering to these guidelines as a protocol to improve outcomes in patients with severe TBI. Similarly, Faul et al.[4] reported that if Brain Trauma Foundation guidelines were followed more routinely, there would be substantial savings in annual medical and rehabilitation costs, $262 million and $43 million, respectively. Whitmore et al.[5] argued for aggressive treatment of severe TBI based on their cost-effective analysis. In their decision-analytic model, the researchers found that for the average 20 year-old, aggressive care yields a highly significant difference in quality-adjusted life years gained ($p<0.0001$).

Some recent studies have sought to classify the burden of TBI, using International Classification of Diseases 9th Revision, Clinical Modification (ICD-9-CM) Codes. The CDC regularly updates a TBI report, using specific ICD-9-CM codes to identify all TBIs in the United States.1 While Modification (ICD-9-CM) Codes. The CDC regularly updates a TBI report, using specific ICD-9-CM codes to identify all TBIs in the United States.[1] While it stratifies TBI events by emergency department visits, hospitalizations and deaths, it does not explore the economic ramifications of TBI. Hu et al.[6] used the Thomson Reuters MarketScan® database to describe the demographic characteristics, injury severity, morbidity and other variables related to TBI. Using the same ICD-9 codes as in the CDC report, the researchers examined TBI occurrence and mortality rates, and quantified the cost of TBI from the perspective of payers (insurance companies). In a 2008 retrospective review of medical records, Carroll et al.[7] sought to understand the sensitivity of ICD-9 codes in the definition of TBI. They found the ICD-9 TBI codes to be 89% sensitive for the presence of any TBI compared with medical records (specificity was not determined because all cases had TBI). Despite this research, the literature often discusses TBI without differentiating between treatment settings, making it difficult to understand the factors associated with TBIs requiring different levels of care. Furthermore, there is little evidence on the hospital costs associated with TBI.[8]

To bridge these gaps in the literature, this study sought to estimate hospital-reported rates and costs associated with TBI. TBI visits were stratified by inpatient and outpatient visits. The study examined the corresponding patient characteristics, hospital demographics, healthcare utilization and trends over time of TBI visits from the perspective of the hospital.

### METHODS

This study utilized clinical and billing data from the Premier hospital database.[9] This database contains clinical and utilization information from more than 600 US hospitals and ambulatory surgery centers. It includes over 45 million inpatient discharges and more than 210 million hospital outpatient visits from acute care facilities, ambulatory surgery centers and clinics across the nation. The data used in this study included hospital discharge data from January 1, 2007 through December 31, 2012. Only hospitals (284 hospitals) included in all 6 years of the analysis time frame were used to identify TBI visits.

Patient visits were identified by the presence of one or more TBI-related ICD-9 codes during the time of their visit. These codes were: 800.xx – 804.xx; 850.1 – 850.5; 850.9; 851.xx – 854.xx; and 959.01. These codes are consistent with the literature defining TBI.[1,6,7] Patient characteristics examined included age (<18, 18-24, 25-44, 45-64, 65+), race (Caucasian, African-American, other), gender, marital status (married, unmarried, other/ unknown) and insurance type (commercial, Medicaid, Medicare, managed care, other). Patient's health status and mortality risk were assessed by the 3M™ All Patient Refined Diagnosis Related Group (APR-DRG) Health Status and Mortality Status system, a patient risk stratification system that stratifies inpatient visits by how sick the patient was and how likely they were to die during that visit.[10] Health status was categorized as requiring inpatient treatment for minor, moderate, severe or extreme medical problems, or requiring only outpatient care. Similarly, mortality risk was categorized by an inpatient visit with minor, moderate, severe or extreme risk of mortality, or requiring only outpatient care. Patient visits were also examined by admission source (the vast majority were admitted to the emergency room) and discharge status (the vast majority were discharged home or to a home health agency). Hospital characteristics examined included census region, urban or non-urban setting, hospital teaching status and hospital size based upon number of beds (<100, 100-199, 200-299, 300-399, 400-499, 500+).

Descriptive statistics were provided for the 2012 TBI patient visits for patient characteristics, hospital characteristics, length of stay, charges and costs. The year 2012 was chosen to provide the most recent estimates of these statistics. Continuous variables such as patient visits were summarized by means, medians and standard deviations. Binary variables were reported as proportions. T-tests and Chi-square tests were used to determine whether differences in these proportions between inpatient and outpatient TBI visits were statistically significant.

The proportion of visits identified as having a TBI were calculated by dividing the number of new visits of interest by the entire number of hospital visits for a given year. For example, the proportion of TBI visits in 2007 was obtained by dividing the number of TBI visits by the number of Premier Hospital visits for 2007. TBI visit rates were calculated for the following categories: overall TBI visits, inpatient TBI visits, outpatient TBI visits, US census regions (Midwest, Northeast, South, and West) and age (<18, 18-24, 25-44, 45-64, ≥65). Compound Annual Growth Rates (CAGR) were calculated from the reported rates using the following equation:

(1) $$CAGR = (Ending\ Value/Beginning\ Value)^{1/\#\ of\ years} - 1$$

### RESULTS

During our study time frame, 2007 - 2012, 1,544,922 TBI-related patient visits were identified. Table 1 shows the characteristics of the 305,077 TBI patients in our 2012 sample, of whom 36,373 (11.9%), were treated on an inpatient basis and 268,704 (88.1%) were treated as outpatients.

Turning first to inpatient TBI patient visits, Table 1 reveals that these visits were concentrated among older age groups. Indeed, more than 44% of inpatient TBI visits were for patients aged 65 or above. The age distribution is more uniform among TBI outpatient visits, though concentrations among the young (<18 years) and elderly (65 and over) were apparent. Overall TBI visits were concentrated among the very young and the very old as well. Patients in both inpatient and outpatient TBI cohorts were largely Caucasian and unmarried. There were more males than females in each cohort. Medicare insured the largest share of TBI inpatient visits (43.3%), reflecting the relatively older age of this cohort. Insurance status was more evenly distributed across payers in the outpatient TBI visit cohort.

**Table 1.** 2012 TBI Patient Characteristics

| | Overall | | TBI Inpatient | | TBI Outpatient | | |
|---|---|---|---|---|---|---|---|
| Category | N | Percent | N | Percent | N | Percent | P-value |
| **Total N** | **305 077** | **100%** | **36 373** | **100%** | **268 704** | **100%** | |
| **Age** | | | | | | | |
| <18 | 89 063 | 29.19 | 3003 | 8.26 | 86 060 | 32.03 | <0.0001 |
| 18-24 | 35 166 | 11.53 | 3074 | 8.45 | 32 092 | 11.94 | |
| 25-44 | 58 505 | 19.18 | 5829 | 16.03 | 52 676 | 19.6 | |
| 45-64 | 52 430 | 17.19 | 8363 | 22.99 | 44 067 | 16.4 | |
| 65+ | 69 913 | 22.92 | 16 104 | 44.27 | 53 809 | 20.03 | |
| **Race** | | | | | | | |
| Caucasian | 210 606 | 69.03 | 26 662 | 73.3 | 183 944 | 68.46 | <0.0001 |
| African American | 42 080 | 13.79 | 3312 | 9.11 | 38 768 | 14.43 | |
| Other | 52 391 | 17.17 | 6399 | 17.59 | 45 992 | 17.12 | |
| **Gender** | | | | | | | |
| Female | 145 124 | 47.57 | 15 034 | 41.33 | 130 090 | 48.41 | <0.0001 |
| Male | 159 953 | 52.43 | 21 339 | 58.67 | 138 614 | 51.59 | |
| **Marital Status** | | | | | | | |
| Married | 64 910 | 21.28 | 10 886 | 29.93 | 54 024 | 20.11 | <0.0001 |
| Unmarried | 211 166 | 69.22 | 21 616 | 59.43 | 189 550 | 70.54 | |
| Other/Unknown | 29 001 | 9.51 | 3871 | 10.64 | 25 130 | 9.35 | |
| **Insurance** | | | | | | | |
| Commercial | 28 636 | 9.39 | 3640 | 10.01 | 24 996 | 9.30 | <0.0001 |
| Medicare | 73 412 | 24.06 | 15 761 | 43.33 | 57 651 | 21.46 | |
| Medicaid | 59 958 | 19.65 | 4074 | 11.20 | 55 884 | 20.80 | |
| Managed Care | 70 010 | 22.95 | 5433 | 14.94 | 64 577 | 24.03 | |
| Other | 73 061 | 23.95 | 7465 | 20.52 | 65 596 | 24.41 | |
| **3M™ APR DRG Health Status** | | | | | | | |
| Minor | 6380 | 2.09 | 6380 | 17.54 | 0 | 0 | <0.0001 |
| Moderate | 13 149 | 4.31 | 13 149 | 36.15 | 0 | 0 | |
| Major | 11 358 | 3.72 | 11 358 | 31.23 | 0 | 0 | |
| Extreme | 5486 | 1.8 | 5486 | 15.08 | 0 | 0 | |
| Outpatient | 268 704 | 88.08 | 0 | 0 | 268 704 | 100 | |
| **3M™ APR DRG Mortality Status** | | | | | | | |
| Minor | 14 820 | 4.86 | 14 820 | 40.74 | 0 | 0 | <0.0001 |
| Moderate | 10 271 | 3.37 | 10 271 | 28.24 | 0 | 0 | |
| Major | 6432 | 2.11 | 6432 | 17.68 | 0 | 0 | |
| Extreme | 4850 | 1.59 | 4850 | 13.33 | 0 | 0 | |
| Outpatient | 268 704 | 88.07 | 0 | 0 | 268 704 | 100 | |
| **Admission Source** | | | | | | | |
| Emergency | 188 869 | 61.91 | 24 637 | 67.73 | 164 232 | 61.12 | <0.0001 |
| Urgent | 19 649 | 6.44 | 4 622 | 12.71 | 15 027 | 5.59 | |
| Elective | 16 313 | 5.35 | 1317 | 3.62 | 14 996 | 5.58 | |
| New Born | 26 | 0.01 | 26 | 0.07 | 0 | 0 | |
| Trauma Center | 7123 | 2.33 | 5446 | 14.97 | 1677 | 0.62 | |
| Unknown | 73 097 | 23.96 | 325 | 0.89 | 72 772 | 27.08 | |

**Table 1.** 2012 TBI Patient Characteristics - Cont.

| | Overall | | TBI Inpatient | | TBI Outpatient | | |
|---|---|---|---|---|---|---|---|
| **Category** | **N** | **Percent** | **N** | **Percent** | **N** | **Percent** | **P-value** |
| **Total N** | **305 077** | **100%** | **36 373** | **100%** | **268 704** | **100%** | |
| **Discharge Status** | | | | | | | |
| Death | 2510 | 0.82 | 2119 | 5.83 | 391 | 0.15 | <0.0001 |
| Home / Home Health | 271 127 | 88.87 | 21 744 | 59.78 | 249 383 | 92.81 | |
| Hospice | 897 | 0.29 | 799 | 2.2 | 98 | 0.04 | |
| Hospital | 1671 | 0.55 | 414 | 1.14 | 1257 | 0.47 | |
| Other | 15 988 | 5.24 | 1590 | 4.37 | 14 398 | 5.36 | |
| Rehab Facility | 3601 | 1.18 | 3527 | 9.7 | 74 | 0.03 | |
| Skilled Nursing | 9283 | 3.04 | 6180 | 16.99 | 3103 | 1.15 | |

TBI: traumatic brain injury; APR DRG: All Patient Refined Diagnosis Related Group

According to the 3M$^{TM}$ APR DRG stratification system, most inpatient TBI visits had moderate (36.15%) or major (31.23%) health status problems, and the majority of these inpatient visits were at minor (40.74%) or moderate (28.24%) mortality risk.

The majority of inpatient (67.73%) and outpatient (61.12%) patients were admitted through the emergency department. However, a significant proportion of inpatient TBI patients (14.97%) were admitted directly to a trauma center. Most patients with TBI-related inpatient visits (59.78%) were discharged to their homes or to a home health agency. The vast majority of patients with outpatient TBI visits (92.81%) were also discharged home or to a home health agency. Nearly 27% of patients requiring inpatient treatment were discharged to a rehab facility or skilled nursing facility. Almost 6% of inpatient TBI visits resulted in death at the time of the visit. This figure is just 0.15% for outpatient TBI visits.

Supplementary Table 1 shows the characteristics of hospitals treating TBI patients by location, teaching status and size. These hospitals were concentrated in the South US Census Region and in urban locations. The majority of hospitals treating TBIs were teaching facilities and tended to be larger hospitals, with 200 beds or more. Inpatient TBI visits were concentrated in very large hospitals — 43.77% of these patients were treated in hospitals having 500 or more beds.

Table 2 shows utilization, charge, and cost information. The median length of stay (LOS) for a TBI inpatient visit was 3 days and the average length of stay 4.76 days (Supplementary Figure 1). Mean hospital inpatient charges were more than $53,000, well above median charges of $34,231, suggesting that a relatively small percentage of inpatients required particularly intensive treatment. Inpatient costs were far below hospital charges for the visit, however, with average costs of $12,717 and median costs of $8,176. The average TBI inpatient visit cost per day and median cost per day were $3,206 and $2,521, respectively. As Table 2 indicates, charges and costs were much lower for TBI patients in the outpatient setting.

**Table 2.** 2012 TBI Visits - Length of Stay, Charges and Costs

| Category | Statistics | Overall | TBI Inpatient | TBI Outpatient | P-value |
|---|---|---|---|---|---|
| **Total** | | 305 077 | 36 373 | 268 704 | |
| **Length of Stay (days)** | Median | | 3 | | |
| | Mean | | 4.76 | | |
| | St. dev. | | 4.83 | | |
| **Total Hospital Charges** | Median | $3517 | $34 231 | $3042 | |
| | Mean | $9920 | $53 199 | $4276 | <0.0001 |
| | St. dev. | $25 498 | $57 660 | $5028 | |
| **Total Hospital Costs** | Median | $497 | $8176 | $435 | |
| | Mean | $2054 | $12 717 | $663 | <0.0001 |
| | St. dev. | $5829 | $12 586 | $998 | |
| **Hospital Costs Per Day** | Median | | $2521 | | |
| | Mean | | $3206 | | |
| | St. dev. | | $2753 | | |

TBI: traumatic brain injury

Figure 1 shows overall trends in proportion of TBI visits. The rate of TBI inpatient visits increased from 0.89% in 2007 to 0.99% by 2012 (CAGR=5.4%) within the Premier hospitals included in this study. The outpatient TBI visit rate grew more rapidly, from 0.73% in 2007 to 0.98% by 2012 (CAGR=6%). Supplementary Figures 2-3 show trends in TBI visits by Census Region of hospital location and patient age, respectively. Supplementary Figure 2 reveals that TBI visits have trended upward in all Census Regions, but has shown a particularly rapid increase in the Northeast Census Region, growing from 0.79% in 2007 to 1.25% by 2012 (CAGR=9.6%). Supplementary Figure 3 indicates that rates of TBI are highest for children (e.g., <18 years of age) and that the rate rose rapidly for this cohort (CAGR=6.6%). The proportion of visits with a TBI was lowest among subjects aged 45 to 64 at the time of visit. The TBI growth rate was lowest for the 18 to 24 age cohort (CAGR=4%).

**Figure 1.** TBI Incidence Rates by Site of Care

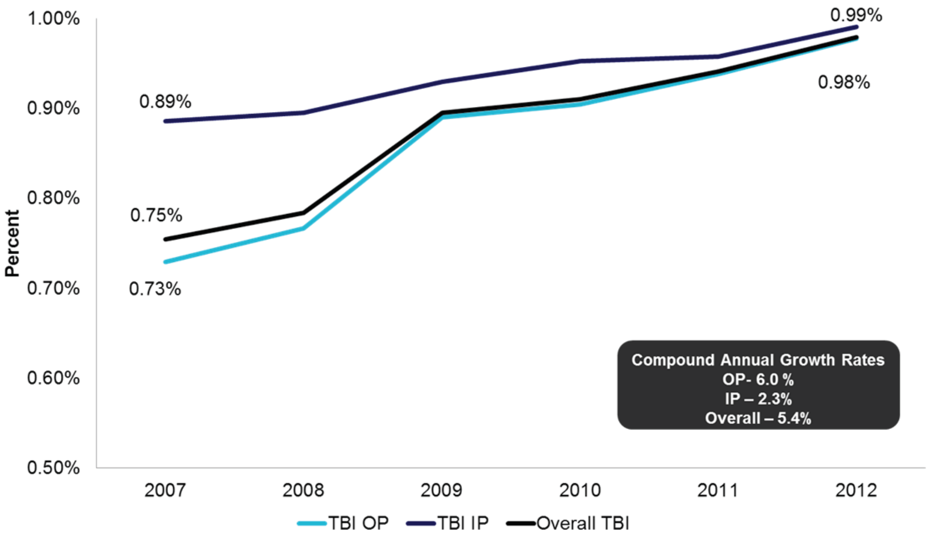

The incidence rate was calculated by dividing the number of outpatient/inpatient/overall TBI visits by the total number of outpatient/inpatient/overall visits in Premier data. TBI: traumatic brain injury; OP: outpatient; IP: inpatient

### DISCUSSION

This descriptive study sought to provide evidence on the characteristics of TBI patient visits, utilization, charges, TBI-related hospital costs and trends in TBI visit rates. Previous studies[6,8] have not distinguished TBI patients by inpatient and outpatient visits. Further, these studies have not examined the costs associated with a TBI visit incurred by the hospital.

This study found that the overall median cost to a hospital of a TBI inpatient visit was $8,176 and the cost of a TBI outpatient visit was $435. The overwhelming number of TBI visits (88%) were treated on an outpatient basis, reducing the overall median hospital cost to $497. The large disparity in costs between inpatient admissions and outpatient visits was not surprising. However, it provided evidence that aggregating all TBI visits together obscures the resources necessary to treat TBIs requiring an inpatient admission.

Hu et al. found the median insurer cost of a TBI inpatient admission to be $10,694.[6] However, this study was conducted from a payer perspective rather than the hospital perspective. This difference in the perspective accounts for the substantial variance in inpatient costs between that study and ours.

To examine inpatient costs of a TBI admission in context with hospital costs of other inpatient admissions, we compared our results to overall inpatient admission costs and those for intracranial injury compiled by the Healthcare Cost and Utilization Project (HCUP) of the Agency for Healthcare Research and Quality (AHRQ).[10] In 2010, the average cost of all hospital inpatient stays was $9,700.[11] The average cost of a stay for intracranial injury inpatient admission was $18,000. AHRQ defined intracranial injury using Clinical Classification Software (CCS).[11] The CCS definition of intracranial injury differs from the definition of TBI used in this study. Our definition, consistent with the CDC's definition, was broader than that used by AHRQ and resulted in average TBI-related inpatient hospital costs of $12,717. Nonetheless, our study suggests the hospital costs associated with TBI were substantial. Indeed, the estimated annual aggregate hospital cost of TBI visits was $3.5 billion (overall average cost of a TBI visit [inpatient and outpatient], $2,054, multiplied by the CDC's8 estimate of annual United States TBI visits, 1.7 million).

This study found a particularly high rate of inpatient TBI visits among elderly patients aged 65 and over and the young, under age 18 years. In addition, the average hospital cost of a TBI visit was $12,623 and $9,930 for elderly patients and the young, respectively. A higher rate of falls or other accidents among older patients, in conjunction with other comorbidities may have played an important factor here. More research into the causes of TBI among the elderly would shed important light on this issue and potentially aid in the development of strategies to prevent these TBIs. Children were most likely to be treated as an outpatient and had an inpatient LOS of 3.2 days compared to 4.8 days for all TBI inpatient visits, suggesting the initial injury was less severe. However, ascertaining the causes of these events and understanding if the young are having multiple TBI visits is an important direction for future study. This avenue of research may lead to improvements in the prevention and treatment of TBI.

There was considerable variation in health status and mortality risk among TBI patients treated on an inpatient basis. This suggests that the burden of TBI may vary considerably among inpatients. Classification systems such as the 3M$^{TM}$ APR-DRG Health Status and Mortality Status system provide a fairly precise severity adjustment of the patient based on their underlying conditions.[10] But more detailed severity adjustments and multivariable analysis of TBI inpatient visits by the patient's health status and mortality risk should further clarify the health and cost consequences of TBI and provide useful inputs for efforts to model the cost and cost-effectiveness of alternative treatment modalities for TBI patients.

There are several limitations to this study that must be noted. This was a retrospective analysis using a hospital claims database (Premier). Detailed information about patient characteristics such as lab values, vital signs and neurological exams were not available. For instance, it would have been interesting to examine severity of TBI through the use of the Glasgow Coma Score, which is a neurological scale. Further, the Premier hospital database may be subject to errors of omission in the coding of TBI. The Premier hospital database is not representative of all US hospitals; therefore, caution should be taken in generalizing these findings. However, the 284 hospitals included in the analysis represent nearly 5% of US hospitals and are well distributed across the country mitigating this concern. Additionally, it was not possible to ascertain why the rates of TBI visits have risen fairly sharply over time, given the data source. Finally, the Premier database does not allow for tracking patients over time or through all avenues of health care providers. Thus, it was not possible to fully assess the number of TBI patients who sought follow-up care at a later date or with their primary care physician.

### CONCLUSION

TBI is a serious medical condition that appears to be on the rise. To date, most research using claims data has focused on all TBI cases and has not examined the significant differences that may exist between TBI treatment in the inpatient and outpatient setting. Future research using large-sample claims data should further stratify TBI to better understand the factors affecting the costs and clinical outcomes of TBI. This stratification may inform and refine treatment strategies to enhance patient outcomes while providing cost effective care.

### CONFLICT OF INTEREST DECLARATION

This study was funded by GE Healthcare. PP and DK are current paid employees of GE Healthcare and BP was a paid employee of GE Healthcare at the time of the research. PP and DK declare stock/stock options in GE Healthcare. PM is an employee of CTI Clinical Trial and Consulting Services, a paid consultant of GE Healthcare. JR is a paid consultant of CTI Clinical Trial and Consulting Services. MV has no conflict to disclose.

## APPENDICES

**Supplementary Table 1.** 2012 Hospital Characteristics Associated with TBI Visits

| | Hospital Characteristics | | | | | | | | | | | |
|---|---|---|---|---|---|---|---|---|---|---|---|---|
| | Overall | | | | TBI Inpatient | | | | TBI Outpatient | | | |
| | By Visit | | By Hopsital | | By Visit | | By Hospital | | By Visit | | By Hospital | |
| **Category** | **N** | **%** | **N** | **%** | **N** | **%** | **N** | **%** | **N** | **%** | **N** | **%** |
| **Total N** | 305 077 | 100% | 284 | 100% | 36 373 | 100% | 283 | | 268 704 | 100% | 254 | 100% |
| **Census Region** | | | | | | | | | | | | |
| Northeast | 43 763 | 14.34 | 43 | 15.14 | 8633 | 23.73 | 43 | 15.19 | 35 130 | 13.07 | 22 | 8.66 |
| Midwest | 60 014 | 19.67 | 61 | 21.48 | 7181 | 19.74 | 61 | 21.55 | 52 833 | 19.66 | 58 | 22.83 |
| South | 133 569 | 43.78 | 111 | 39.08 | 13 512 | 37.15 | 111 | 39.22 | 120 057 | 44.68 | 107 | 42.13 |
| West | 67 731 | 22.2 | 69 | 24.3 | 7047 | 19.37 | 68 | 24.03 | 60 684 | 22.58 | 67 | 26.38 |
| **Location** | | | | | | | | | | | | |
| Urban | 270 478 | 88.66 | 226 | 79.58 | 33 923 | 93.26 | 225 | 79.51 | 236 555 | 88.04 | 200 | 78.74 |
| Not Urban | 34 599 | 11.34 | 58 | 20.42 | 2450 | 6.74 | 58 | 20.49 | 32 149 | 11.96 | 54 | 21.26 |
| **Type** | | | | | | | | | | | | |
| Teaching | 104 007 | 34.09 | 70 | 24.65 | 20 099 | 55.26 | 70 | 24.73 | 83 908 | 31.23 | 58 | 22.83 |
| Non-Teaching | 201 070 | 65.91 | 214 | 75.35 | 16 274 | 44.74 | 213 | 75.27 | 184 796 | 68.77 | 196 | 77.17 |
| **Bed Count** | | | | | | | | | | | | |
| <100 | 19 810 | 6.49 | 47 | 16.55 | 518 | 1.42 | 46 | 16.25 | 19 292 | 7.18 | 43 | 16.93 |
| 100-199 | 37 992 | 12.45 | 55 | 19.37 | 2089 | 5.74 | 55 | 19.43 | 35 903 | 13.36 | 47 | 18.5 |
| 200-299 | 55 366 | 18.15 | 60 | 21.13 | 5968 | 16.41 | 60 | 21.2 | 49 398 | 18.38 | 54 | 21.26 |
| 300-399 | 64 859 | 21.26 | 52 | 18.31 | 6156 | 16.92 | 52 | 18.37 | 58 703 | 21.85 | 48 | 18.9 |
| 400 - 499 | 49 015 | 16.07 | 34 | 11.97 | 5722 | 15.73 | 34 | 12.01 | 43 293 | 16.11 | 30 | 11.81 |
| >500 7 | 8,035 | 25.58 | 36 | 12.68 | 15 920 | 43.77 | 36 | 12.72 | 62 115 | 23.12 | 32 | 12.6 |

TBI: traumatic brain injury

**Supplementary Figure 1.** Length of Stay of 2012 Inpatient TBI Visits – days

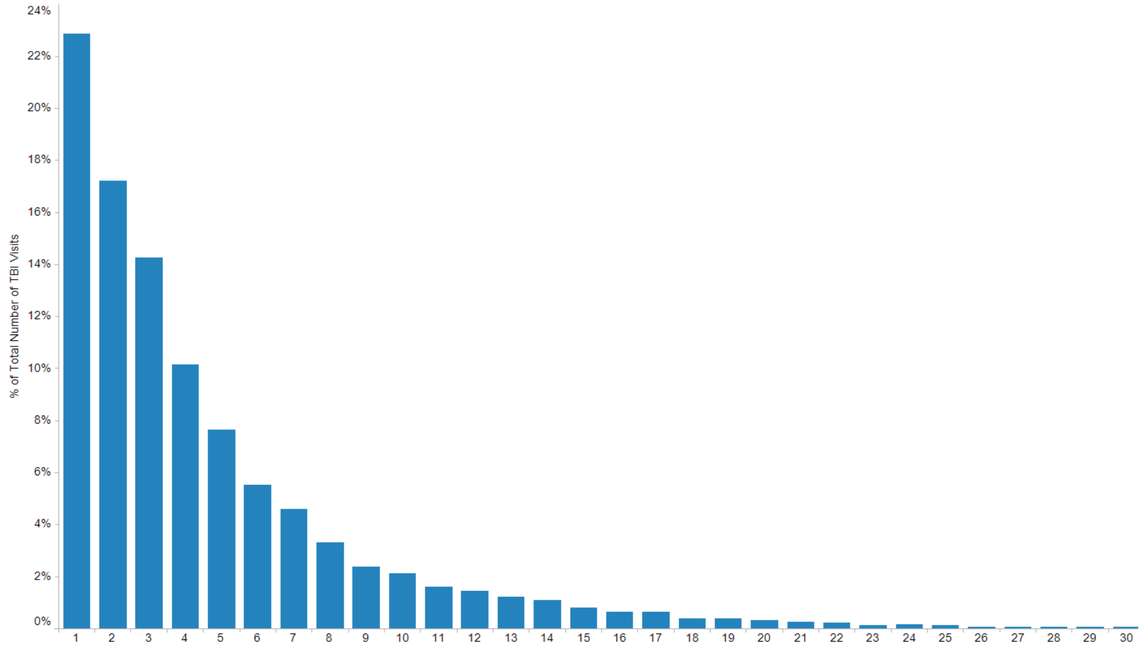

**Supplementary Figure 2.** TBI Incidence Rates by Census Region

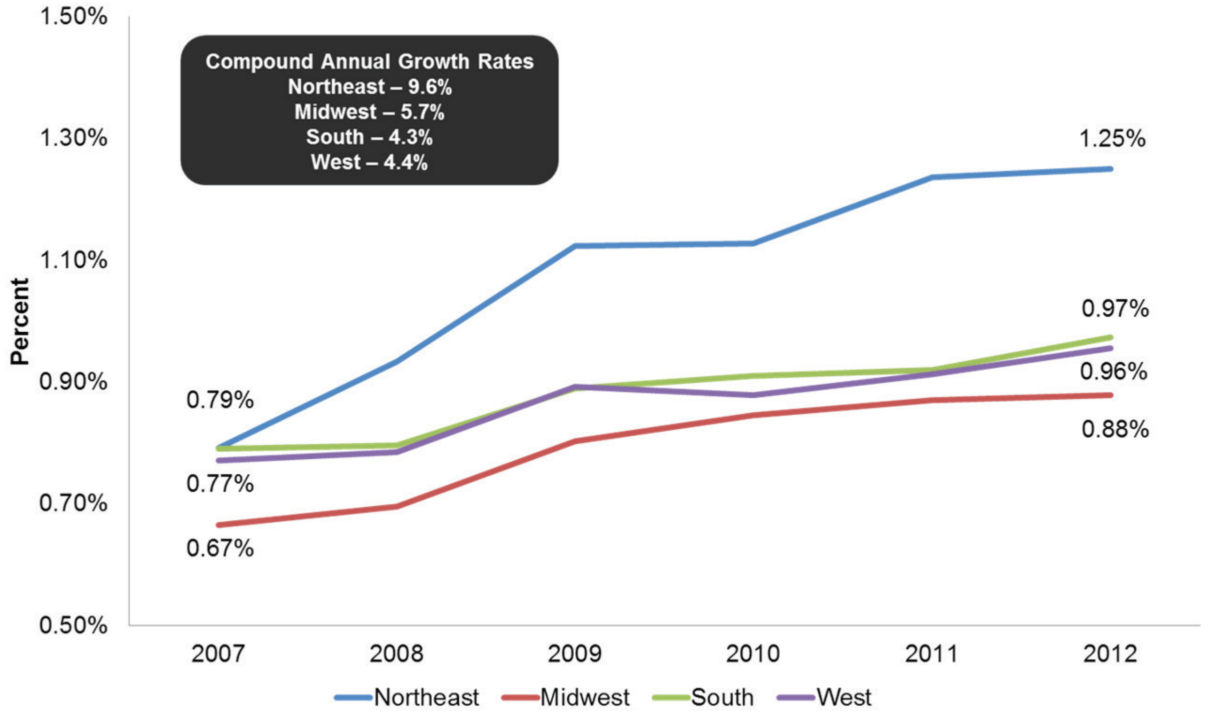

**Supplementary Figure 3.** TBI Incidences Rates by Age Cohort

## References

[1] Faul M, Xu L, Wald MM, et al: Traumatic brain injury in the United States: Emergency department visits, hospitalizations and deaths 2002–2006. Atlanta, GA: Centers for Disease Control and Prevention, National Center for Injury Prevention and Control, 2010.

[2] Finkelstein E, Corso PS, Miller TR: The incidence and economic burden of injuries in the United States. Oxford ; New York: Oxford University Press; 2006.

[3] Hesdorffer DC, Ghajar J: Marked improvement in adherence to traumatic brain injury guidelines in United States trauma centers. *J Trauma* 2007;63(4):841-7; discussion 7-8.

[4] Faul M, Wald MM, Rutland-Brown W, et al: Using a cost-benefit analysis to estimate outcomes of a clinical treatment guideline: testing the Brain Trauma Foundation guidelines for the treatment of severe traumatic brain injury. *J Trauma* 2007;63(6):1271-8.

[5] Whitmore RG, Thawani JP, Grady MS, et al: Is aggressive treatment of traumatic brain injury costeffective? *J Neurosurg* 2012;116(5):1106-13.

[6] Hu J, Ugiliweneza B, Meyer K, et al: Trend and geographic analysis for traumatic brain injury mortality and cost based on MarketScan database. *J Neurotrauma* 2013;30(20):1755-61.

[7] Carroll CP, Cochran JA, Guse CE, et al: Are we underestimating the burden of traumatic brain injury? Surveillance of severe traumatic brain injury using centers for disease control International classification of disease, ninth revision, clinical modification, traumatic brain injury codes. *Neurosurgery* 2012;71(6):1064-70; discussion 70.

[8] Centers for Disease Control and Prevention: Injury and prevention Control: Traumatic Brain Injury. 2014 http://www.cdc.gov/traumaticbraininjury/statistics.html. Accessed July 2014.

[9] Premier Research Services 2013. http://www.premierinc.com. Accessed January 15, 2014.

[10] 3M™ APR DRG Classification System and 3M™ APR DRG Software.

[11] Pfuntner A, Wier LM, Steiner C: Costs for hospital stays in the United States, 2010. HCUP Statistical Brief #146. Rockville, MD: Agency for Healthcare Research and Quality; January 2013.

[12] Healthcare Cost and Utilization Project (HCUP). Clinical classifications software (CSS) for ICD-9- CM. January 2014. www.hcup-us.ahrq.gov/toolssoftware/ccs/ccs.jsp. Accessed January 14, 2014.
